# Evaluation and Comparison of Capillary Morphology Among Combusted Tobacco, Heated Tobacco and E-Cigarette Users

**DOI:** 10.3390/biology14020163

**Published:** 2025-02-06

**Authors:** Salvatore Nigliaccio, Davide Alessio Fontana, Antonino Cacioppo, Luciano Curcio, Enzo Cumbo, Giuseppe Alessandro Scardina, Pietro Messina

**Affiliations:** 1 Department of Precision Medicine in Medical, Surgical and Critical Care (Me.Pre.C.C.), University of Palermo, 90128 Palermo, Italy; salvo.nigliaccio@gmail.com (S.N.); davidealessiofontana@libero.it (D.A.F.); cacioppoantonino@gmail.com (A.C.); enzo.cumbo@unipa.it (E.C.); pietro.messina01@unipa.it (P.M.); 2 Department of Earth and Marine Sciences (DiSTeM), University of Palermo, 90123 Palermo, Italy; luciano.curcio@unipa.it

**Keywords:** videocapillaroscopy, combusted tobacco, heated tobacco, electronic cigarette

## Abstract

The habit of smoking in its various forms represents a significant public health concern due to its wide range of pathological effects, included the oral cavity. In recent years, alternatives to traditional cigarettes, such as heated tobacco products and electronic cigarettes, have gained popularity and are often marketed as potentially less harmful options. This study seeks to evaluate and compare the morphometric characteristics of oral mucosal capillaries in individuals who consume combusted tobacco, heated tobacco, vaporized liquid, and non-smokers. Using videocapillaroscopy, we assessed both parametric and non-parametric data from 60 patients, divided into four groups according to their smoking habits. The analysis revealed significant differences in capillary morphology among the groups. Users of combusted tobacco exhibited pronounced reductions in capillary diameter, alongside increased tortuosity and the presence of microaneurysms. These alterations are indicative of chronic inflammation and vasoconstriction, likely driven by exposure to nicotine and the high temperatures associated with combustion. Conversely, users of heated tobacco and vaporized liquid exhibited comparatively fewer vascular abnormalities, although angiogenic effects attributable to nicotine were still observable. These findings suggest that alternative tobacco products may have a comparatively lesser impact on the oral microcirculation when compared to traditional smoking. However, the potential long-term effects of these products remain unclear. Further longitudinal research is required to fully understand the risks associated with prolonged use of heated tobacco and electronic cigarettes.

## Simple Summary

This study investigates how different types of smoking, including conventional cigarettes, heated tobacco, and e-cigarettes, affect the capillaries in the oral cavity. While the harmful effects of traditional tobacco use are well-documented, the risks posed by newer forms of smoking remain less clear, particularly regarding their impact on oral health. The primary aim of this research was to compare the effects of these different tobacco products on the microvascular structure of the oral mucosa. The results revealed distinct differences in vascular characteristics, with traditional cigarette smokers showing the most significant changes. These findings are crucial for understanding the specific risks associated with both conventional and newer tobacco products, helping to fill the gap in knowledge regarding the oral health consequences of e-cigarettes and heated tobacco. The study’s outcomes could inform clinical practices aimed at preventing tobacco-related oral diseases and guide public health efforts to raise awareness about the broader health risks of all tobacco products, including those considered to be less harmful than conventional cigarettes.

## 1. Introduction

The habit of smoking, whether through traditional cigarettes, heated tobacco products, or vaporized liquids, continues to pose a significant public health challenge due to its widespread prevalence and numerous adverse health effects. Smoking has been unequivocally linked to a range of systemic and localized diseases, including cardiovascular and respiratory conditions, as well as pathological changes in the oral cavity. Specifically, tobacco use in the oral environment is associated with mucosal damage, periodontal disease, and oral cancer [1]. These harmful effects are primarily mediated by toxic chemicals present in tobacco smoke and the high temperatures generated during combustion, which induce both direct and indirect damage to the tissues and microcirculation of the oral cavity [2].

Recent years have witnessed a shift in smoking habits, with an increasing number of individuals, especially young people, transitioning to alternative tobacco products such as heated tobacco devices and electronic cigarettes [3]. These products are frequently promoted as less harmful alternatives to conventional cigarettes, primarily due to differences in their methods of nicotine delivery and the lower temperatures at which they operate.

Combusted tobacco, which involves burning at temperatures exceeding 900 °C, produces smoke containing over 70 carcinogenic substances, including tobacco-specific nitrosamines (TSNAs), polycyclic aromatic hydrocarbons (PAHs), heavy metals, and aromatic amines, in addition to nicotine [4,5,6].

Heated tobacco products, such as IQOS, operate at significantly lower temperatures (around 350 °C) and produce an aerosol containing nicotine and a reduced number of harmful substances [7]. While the levels of certain substances are lower compared to combusted tobacco, these products are not without risk [8].

Electronic cigarettes vaporize a liquid containing nicotine, flavorings, and various chemicals at even lower temperatures. However, the diversity of devices and liquids, as well as the potential for users to customize their mixtures, complicates the standardization and quantification of the chemical composition of the aerosol produced [9,10].

Despite their growing popularity, long-term evidence on the health risks associated with heated tobacco and electronic cigarettes remains limited, particularly regarding their effects on the oral cavity [8,11]. One critical area of investigation is their impact on the oral microcirculation, specifically the capillary network. Alterations in capillary density, caliber, and tortuosity are recognized as early indicators of microvascular damage and can precede more severe pathological conditions. Microcirculatory changes are influenced by immunological and biochemical events, commonly observed in both inflammatory and autoimmune pathologies [12].

Videocapillaroscopy, a non-invasive diagnostic tool, allows for detailed visualization of microvascular structures and has proven effective in detecting and monitoring microvascular alterations in various clinical settings.

For over two decades, the study of oral microcirculation in smokers has been a key area of focus in scientific research. While methodological standardization has often been limited, early studies have consistently demonstrated significant changes in combusted tobacco users, such as reduced capillary caliber, increased density, and greater tortuosity [13]. However, there is currently a lack of comparable research examining these parameters in users of heated tobacco or vaporized liquid products, leaving an important gap in the literature.

The aim of this study is to compare the morphometric characteristics of oral mucosal capillaries in patients who use three different types of smoking: combusted tobacco, heated tobacco, and vaporized liquid. By assessing the capillary morphology using videocapillaroscopy, this study seeks to determine if alternative tobacco products are indeed less harmful to the oral microcirculation compared to conventional cigarettes. This investigation could provide valuable insights into the relative risks associated with these products, contributing to the ongoing debate about their safety.

## 2. Materials and Methods

### 2.1. Study Design and Participants

This experimental study was conducted over a period of 18 months at the Clinical Odontostomatology Department of Università degli Studi di Palermo. A total of 60 participants were recruited and divided into four groups (15 participants each) based on their tobacco consumption habits:

- Combusted tobacco users (cigarette smokers);
- Heated tobacco users (IQOS, Glo, or similar devices);
- Vaporized liquid users (e-cigarette users);
- Non-smokers.

All participants provided written informed consent prior to their enrollment in the study. The anamnesis process was conducted in two distinct phases. The first phase involved the detailed collection of information during the compilation of the medical record, including the patient’s general health status, use of medications, and lifestyle habits. Patients who met the eligibility criteria for the study proceeded to the second phase, which focused specifically on smoking, allowing for an in-depth assessment of all aspects related to tobacco use.

All patients observed a waiting period of at least 30 min between the anamnesis phase and the videocapillaroscopy procedure, during which they refrained from consuming food, beverages (apart from water), or candies.

Participants were selected according to specific inclusion and exclusion criteria to ensure the reliability of the results.

#### 2.1.1. Inclusion Criteria

- Participants aged between 18 and 70 years;
- Continuous use of the specified tobacco product for at least two years was required for inclusion in the smoker groups, while the control group of non-smokers had never used tobacco;
- General good health with no chronic conditions affecting microcirculation;
- No discrimination based on gender.

#### 2.1.2. Exclusion Criteria

- Combined use of multiple forms of tobacco;
- Systemic conditions: diabetes, metabolic syndrome, hypertension, autoimmune diseases, or vascular or rheumatic diseases;
- Pregnancy;
- Medications: regular use of drugs affecting microcirculation;
- Substance abuse: alcohol and drug abuse in quantities that could impact microcirculation.

#### 2.1.3. Demographic and Habitual Characteristics of Study Participants

The mean age of burnt tobacco users was higher (49.8 ± 13.6 years, range 26–68) compared to heated tobacco (43.3 ± 15.7 years, range 25–64), vaporized liquid users (33 ± 10.5 years, range 24–55), and non-smokers (43.8 ± 14.4 years, range 23–61). The duration of the habit was longest in burnt tobacco users (33.3 ± 14.18 years), followed by heated tobacco (3.3 ± 0.86 years) and vaporized liquid users (4.0 ± 1.41 years). The totality of heated tobacco users were cigarette smokers (15 out of 15), while less than the half of the e-cig users were (7 out of 15). Regarding e-cig users, 7% reported using nicotine-free liquids, 13% used low-nicotine liquids (3 mg/mL), 33% used medium-nicotine liquids (9 mg/mL), and 47% used high-nicotine liquids (18 mg/mL).

### 2.2. Data Collection

The videocapillaroscopy examinations were conducted using the Horus HS100 device, with the following technical specs: 150x zoom lens, 640 × 480 pixels resolution, frame rate 120 FPS, monochromatic.

The examination focused on four oral sites:

- Right buccal mucosa;
- Left buccal mucosa;
- Lower labial mucosa;
- Upper labial mucosa.

For each examined site, a video of approximately 20 s was recorded in a single area, consistently targeting the same anatomical landmarks to ensure uniformity in data collection.

### 2.3. Image Processing and Analysis

**Figure 1:**
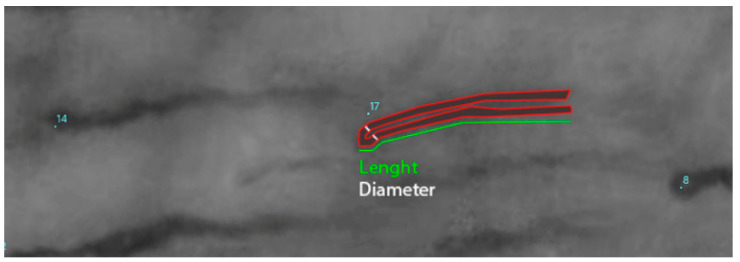
Highlighting of a capillary loop. The number indicates the number of the capillaries.

After the procedure, videos were exported in AVI format and the clearest frames were extracted and then processed to optimize contrast, exposure, and color curves for enhanced visibility. Quantitative analysis was performed using dedicated software (ImageJ, v1.54, NIH) with a conversion scale of 2.65 microns per pixel. The analysis was performed in single-blind (Figure 1).

### 2.4. Parameters Analyzed

- Parametric data:
  ○ Loop density: capillaries per mm^2^;
  ○ Loop caliber: mean of arteriolar and venular diameters;
  ○ Loop length in mm.
- Non-parametric data:
  ○ Loop tortuosity: not crossing (0), one crossing (1), multiple crossings (2), complete distortion (3);
  ○ Capillary visibility: clearly visible (1), poorly visible (2), or not visible (3);
  ○ Orientation in relation to the surface: parallel (A), perpendicular (B), or mixed (AB);
  ○ Presence of microhemorrhages and/or microaneurysms;
  ○ Morphological anomalies.

### 2.5. Statistical Analysis

Statistical analysis was performed by dedicated software (XLSTAT, 2024.4.0.1424, Lumivero). For parametric data an ANOVA test was used to compare the groups, followed by a Tukey pairwise test for post hoc analysis when *p*-values were <0.05.

Non-parametric data were analyzed using independence tests to assess the association between rows and columns of contingency tables. Specifically, the Monte Carlo simulation method was employed (with 5000 iterations) to calculate chi-square statistics and estimate *p*-values.

## 3. Results

### 3.1. Non-Smokers

**Figure 2:**
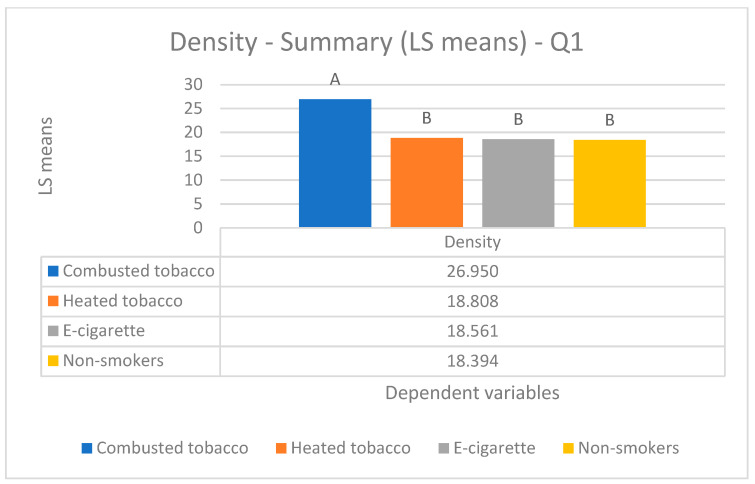
Comparison of capillary density among the four groups enrolled.

**Figure 3:**
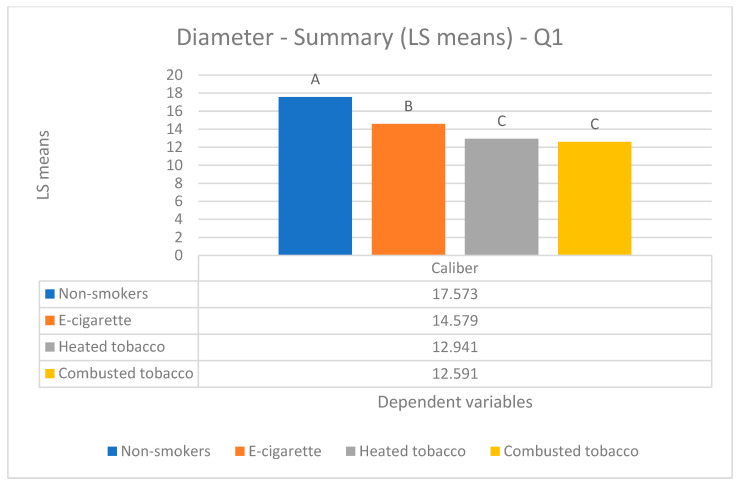
Comparison of capillary diameter among the four groups enrolled.

**Figure 4:**
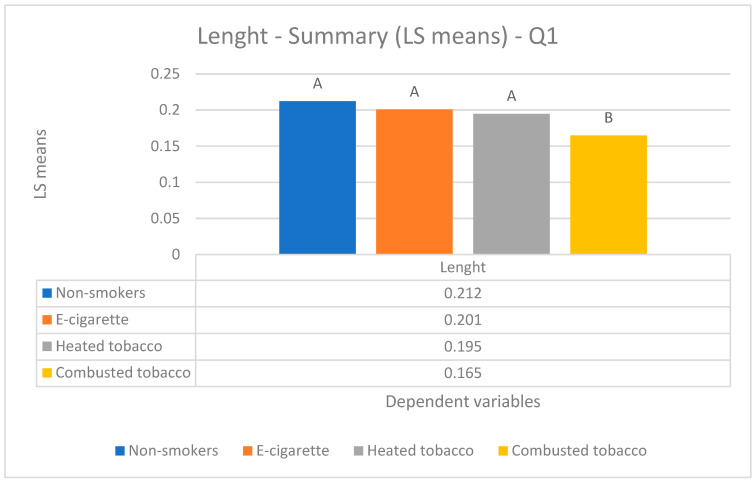
Comparison of capillary lenght among the four groups enrolled.

Parametric Data (mean values) (Figure 2, Figure 3 and Figure 4).

1. Loop density per mm^2^: 18.39 ± 3.53 capillaries/mm^2^;
2. Loop diameter: 17.57 ± 3.03 μm;
3. Loop length: 0.212 ± 0.040 mm.

Non-Parametric Data (Table 1).

1. Loop tortuosity:
  - Not crossing (0): 42;
  - One crossing (1): 9;
  - Multiple crossings (2): 9;
  - Complete distortion (3): 0.
2. Visibility:
  - 58 images were clearly visible (1);
  - 2 images were poorly visible (2);
  - 0 images were not visible (3).
3. Orientation:
  - In 53 images, parallel to the surface (A);
  - In 0 images, perpendicular to the surface (B);
  - In 7 images, both parallel and perpendicular to the surface (AB).
4. Microhemorrhages:
  - No microhemorrhages were observed in 59 images (0);
  - One or more microhemorrhages were observed in 1 image (1), exclusively on the lower labial mucosa.
5. Characteristic loops: Loops with particular morphologies were observed in 0 images.

### 3.2. Combusted Tobacco

Parametric Data (mean values) (Figure 2, Figure 3 and Figure 4).

1. Capillary density per mm^2^: 26.95 ± 9.49 capillaries/mm^2^;
2. Capillary diameter: 12.59 ± 3.92 μm;
3. Loop length: 0.165 ± 0.024 mm.

Non-Parametric Data (Table 1).

1. Loop tortuosity:
  - Not crossing (0): 0;
  - One crossing (1): 4;
  - Multiple crossings (2): 32;
  - Complete distortion (3): 24.
2. Visibility:
  - 40 images were clearly visible (1);
  - 16 images were poorly visible (2);
  - 4 images were not visible (3).
3. Orientation:
  - In 42 images, parallel to the surface (A);
  - In 2 images, perpendicular to the surface (B);
  - In 16 images, both parallel and perpendicular to the surface (AB).
4. Microhemorrhages:
  - No microhemorrhages were observed in 50 images (0);
  - One or more microhemorrhages were observed in 10 images (1), exclusively on the lower labial mucosa.
5. Characteristic loops: Loops with particular morphologies were observed in 22 images. The most common malformations were ectasias, and the most frequent characteristic morphologies were “deer antler” and “cactus” shapes.

### 3.3. Heated Tobacco

Parametric Data (mean values) (Figure 2, Figure 3 and Figure 4).

1. Capillary density per mm^2^: 18.81 ± 7.50 capillaries/mm^2^;
2. Capillary diameter: 12.94 ± 2.67 μm;
3. Loop length: 0.195 ± 0.041 mm.

Non-Parametric Data (Table 1)

1. Loop tortuosity:
  - Not crossing (0): 6;
  - One crossing (1): 8;
  - Multiple crossings (2): 40;
  - Complete distortion (3): 6.
2. Visibility:
  - 50 images were clearly visible (1);
  - 10 images were poorly visible (2);
  - 0 images were not visible (3).
3. Orientation:
  - In 48 images, parallel to the surface (A);
  - In 2 image, perpendicular to the surface (B);
  - In 10 images, both parallel and perpendicular to the surface (AB).
4. Microhemorrhages:
  - No microhemorrhages were observed in 52 images (0);
  - One or more microhemorrhages were observed in 8 images (1): 5 on the lower labial mucosa and 1 on the right buccal mucosa.
5. Characteristic loops: Loops with particular morphologies, as described in the previous chapter, were observed in 18 images. The most common malformations were ectasias, with the “cactus” shape being the most frequent characteristic morphology.

### 3.4. Electronic Cigarette

Parametric Data (mean values) (Figure 2, Figure 3 and Figure 4).

1. Capillary density per mm^2^: 18.56 ± 3.95 capillaries/mm^2^;
2. Capillary diameter: 14.58 ± 2.59 μm;
3. Loop length: 0.201 ± 0.049 mm.

Non-Parametric Data (Table 1)

1. Loop tortuosity:
  - Not crossing (0): 10;
  - One crossing (1): 16;
  - Multiple crossings (2): 29;
  - Complete distortion (3): 5.
2. Visibility:
  - 52 images were clearly visible (1);
  - 8 images were poorly visible (2);
  - 0 images were not visible (3).
3. Orientation:
  - In 44 images, parallel to the surface (A);
  - In 10 images, perpendicular to the surface (B);
  - In 6 images, both parallel and perpendicular to the surface (AB).
4. Microhemorrhages:
  - No microhemorrhages were observed in 59 images (0);
  - One or more microhemorrhages were observed in 1 image (1), located on the right buccal mucosa.
5. Characteristic loops: Loops with particular morphologies, as described in the previous chapter, were observed in 10 images. The most common malformations were ectasias, with the “cactus” shape being the most frequent characteristic morphology.

### 3.5. Comparison

In the analysis of capillary density, statistically significant differences (*p*-value < 0.05) were observed between two groups: one consisting of combusted tobacco users (26.95 ± 9.49 capillaries/mm^2^) and the other including heated tobacco (18.81 ± 7.50 capillaries/mm^2^), e-cigarette users (18.56 ± 3.95 capillaries/mm^2^), and non-smokers (18.39 ± 3.53 capillaries/mm^2^) (Figure 2).

Regarding the diameter of capillary loops, the data revealed statistically significant differences (*p*-value < 0.05) between three groups: one composed of combusted tobacco users (12.59 ± 3.92 μm) and heated tobacco users (12.94 ± 2.67 μm), a separate group of e-cigarette users (14.58 ± 2.59 μm), and another group of non-smokers (17.57 ± 3.03 μm) (Figure 3).

The analysis of loop length revealed a statistically significant difference (*p*-value < 0.05) between two groups: the first one composed of combusted tobacco smokers (0.165 ± 0.024 mm) and the second one composed of heated tobacco (0.195 ± 0.041 mm) and e-cig users (0.201 ± 0.049 mm), as well as non-smokers (0.212 ± 0.040 mm) (Figure 4).

## 4. Discussion

The results of this study provide valuable insights into the differential effects of combusted tobacco, heated tobacco, and vaporized liquids on the oral microcirculation. Through the use of videocapillaroscopy, we documented significant structural and morphometric changes in the capillary network, shedding light on the varying impacts of these products on the oral mucosa.

**Figure 5:**
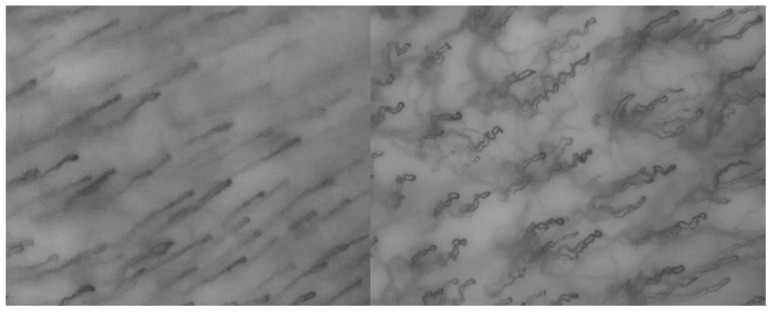
Left cheek mucosa in a non-smoker (**left**) and in a combusted tobacco user (**right**).

Among the groups analyzed, combusted tobacco users exhibited the most pronounced alterations. These included a significant reduction in capillary caliber, increased tortuosity, and a higher prevalence of microaneurysms and capillary crossings (as illustrated in the example shown in Figure 5). These findings are consistent with previous studies highlighting the adverse effects of conventional smoking on the vascular system [13]. Chronic inflammation, persistent vasoconstriction, and endothelial dysfunction are key mechanisms underlying these changes, driven by oxidative stress, the action of reactive oxygen species (ROS), and exposure to toxic compounds such as tobacco-specific nitrosamines (TSNAs), polycyclic aromatic hydrocarbons (PAHs), and heavy metals [14,15]. Additionally, the high combustion temperatures (exceeding 900 °C) not only induce direct thermal damage to oral tissues but also amplify the cytotoxic effects of the inhaled substances, leading to cumulative microvascular injury.

Heated tobacco, while often promoted as a safer alternative to traditional cigarettes, showed moderate vascular alterations. Users of heated tobacco displayed reduced capillary caliber and density compared to vaporized liquid users, although these changes were less severe than those seen in combusted tobacco users. This intermediate effect suggests that while heated tobacco products may mitigate some of the harmful impacts associated with combustion, they are not without risk. The aerosol produced by these devices contains nicotine and various harmful or potentially harmful substances, including acetaldehyde and formaldehyde, albeit in lower concentrations compared to combusted tobacco smoke. Nicotine remains a central player in vascular damage, promoting vasoconstriction, endothelial apoptosis, and inflammatory responses. These findings call for a cautious interpretation of claims regarding the reduced harm of heated tobacco products, particularly in the absence of comprehensive long-term studies.

In contrast, vaporized liquid users demonstrated the least severe capillary alterations, suggesting a relatively lower impact on oral microcirculation. This outcome can be attributed to the absence of combustion and the lower operating temperatures of electronic cigarettes, which likely minimize direct thermal damage. However, it is important to acknowledge that vaporized liquids are not entirely risk-free. The chemical composition of e-liquids and their aerosols varies widely, with many formulations containing potentially harmful substances such as propylene glycol, glycerin, and flavoring agents. While these compounds are generally considered less toxic than those found in combusted tobacco, some studies have shown that they can exert pro-inflammatory and oxidative effects, particularly with prolonged exposure [16]. Furthermore, nicotine delivered via vaping may still induce subtle but clinically relevant changes in the capillary network over time. The lack of standardization in e-liquid formulations and variability in user behaviors further complicate the assessment of the long-term safety of these products.

The gradient of vascular alterations observed across the three groups underscores the interplay between the mode of nicotine delivery, the operating temperatures of the products, and the composition of the inhaled aerosols. Combusted tobacco, with its extreme temperatures and complex toxicant profile, induces the most severe microvascular damage. Heated tobacco, operating at intermediate temperatures, produces less harm but still delivers harmful substances capable of inducing vascular changes. Vaporized liquids appear to exert the least impact, yet the observed alterations indicate that they cannot be considered entirely benign.

Clinically, these findings carry significant implications for understanding and managing smoking-related oral health risks. The capillary alterations observed in combusted tobacco users, such as increased tortuosity and the presence of microaneurysms, may serve as early indicators of systemic microvascular dysfunction. These changes could have downstream effects on tissue oxygenation, wound healing, and susceptibility to oral pathologies, including periodontal disease. Conversely, the relatively preserved capillary morphology in vaporized liquid users suggests a potentially lower risk to oral microcirculation, though further studies are necessary to substantiate this observation.

Moreover, these results highlight the potential of videocapillaroscopy as a non-invasive tool for monitoring microvascular health in smokers. The accessibility of the oral mucosa makes it a practical site for tracking the progression of microvascular changes and evaluating the impact of smoking cessation or product substitution. Longitudinal studies employing this technique could provide crucial insights into the reversibility of smoking-induced vascular damage and the relative safety of alternative tobacco products.

Nevertheless, certain limitations of this study must be acknowledged. To better understand the impact of heated tobacco and vaporized liquids on oral microcirculation, it is essential to study individuals who exclusively use these products without a history of combusted tobacco use. Previous smoking can mask or amplify vascular alterations, making it difficult to isolate the effects of these alternatives [17].

By excluding former smokers, researchers can determine whether the observed changes are directly caused by heated tobacco or vaporized liquids or are remnants of past smoking. Additionally, studying exclusive users can clarify whether these changes progress over time or stabilize, providing valuable insights into the long-term safety of these products.

This approach would not only enhance scientific understanding but also provide clearer data for public health policies, helping to distinguish between genuine harm reduction potential and marketing claims. Additionally, the variability in e-liquid compositions and the reliance on self-reported smoking habits introduce potential sources of bias. Future research should address these limitations by employing larger and more diverse cohorts, as well as standardized e-liquid formulations, to enhance the reliability and comparability of data.

In conclusion, this study sheds light on the differential effects of combusted tobacco, heated tobacco, and vaporized liquids on oral microcirculation. While alternative products appear to pose a reduced risk compared to conventional cigarettes, they are not without consequences. The long-term safety of these products remains uncertain, necessitating further research to inform evidence-based public health policies and clinical guidelines for smoking cessation and harm reduction.

## 5. Conclusions

This study highlights the differential impact of combusted tobacco, heated tobacco, and vaporized liquids on the oral microcirculation. Combusted tobacco exerts the most significant damage, characterized by reduced capillary caliber, increased tortuosity, and greater microvascular abnormalities. Heated tobacco and vaporized liquids show comparatively less severe effects but are not without risks, as nicotine and other substances still contribute to vascular alterations. The findings underscore the importance of further research, particularly with exclusive users of alternative products, to better understand their long-term safety. This knowledge is critical for guiding public health strategies and clinical recommendations for harm reduction and smoking cessation.

## Figures and Tables

**Table 1:** Comparison of non-parametric data. * Indicates a significant value (*p*-value < 0.05).

	Non-Smokers		Combusted Tobacco		Heated Tobacco		E-Cigarette	
Loop tortuosity								
*Not crossing (0)*	42 *	70%	0	0%	6	10%	10	16.7%
*One crossing (1)*	9	15%	4	6.7%	8	13.3%	16 *	26.7%
*Multiple crossings (2)*	9 *	15%	32	53.3%	40 *	66.7%	29	48.3%
*Complete distortion (3)*	0	0%	24 *	40%	6	10%	5	8.3%
Visibility								
*Clearly visible (1)*	58	96.7%	40	66.6%	50	83.3%	52	86.7%
*Poorly visible (2)*	2 *	3.3%	16 *	26.6%	10	16.7%	8	13.3%
*Not visible (3)*	0	0%	4 *	6.6%	0	0%	0	0%
Orientation								
*Parallel (A)*	53	88.3%	42	70%	48	80%	44	73.3%
*Perpendicular (B)*	0	0%	2	3.3%	2	3.3%	10 *	16.7%
*Mixed (AB)*	7	11.7%	16 *	26.7%	10	16.7%	6	10%
Microhemorrhages								
*No (0)*	59	98.3%	50	83.3%	52	86.7%	59	98.3%
*Yes (1)*	1	1.7%	10 *	16.7%	8	13.3%	1	1.7%

## Data Availability

The raw data supporting the conclusions of this article will be made available by the authors on request.

## References

[1] Taybos G. (2003). Oral changes associated with tobacco use. Am. J. Med. Sci..

[2] Borgerding M., Klus H. (2005). Analysis of complex mixtures—Cigarette smoke. Exp. Toxicol. Pathol..

[3] Yoong S.L., Stockings E., Chai L.K., Tzelepis F., Wiggers J., Oldmeadow C., Paul C., Peruga A., Kingsland M., Attia J. (2018). Prevalence of electronic nicotine delivery systems (ENDS) use among youth globally: A systematic review and meta-analysis of country level data. Aust. N. Z. J. Public Health.

[4] Centers for Disease Control and Prevention (US), National Center for Chronic Disease Prevention and Health Promotion (US), Office on Smoking and Health (US) (2010). 3 Chemistry and Toxicology of Cigarette Smoke and Biomarkers of Exposure and Harm. How Tobacco Smoke Causes Disease: The Biology and Behavioral Basis for Smoking-Attributable Disease: A Report of the Surgeon General.

[5] Ji H., Jin Z. (2022). Analysis of six aromatic amines in the mainstream smoke of tobacco products. Anal. Bioanal. Chem..

[6] Caruso R.V., O’Connor R.J., Stephens W.E., Cummings K.M., Fong G.T. (2013). Toxic metal concentrations in cigarettes obtained from U.S. smokers in 2009: Results from the International Tobacco Control (ITC) United States survey cohort. Int. J. Environ. Res. Public Health.

[7] Upadhyay S., Rahman M., Johanson G., Palmberg L., Ganguly K. (2023). Heated Tobacco Products: Insights into Composition and Toxicity. Toxics.

[8] Sever E., Božac E., Saltović E., Simonić-Kocijan S., Brumini M., Glažar I. (2023). Impact of the Tobacco Heating System and Cigarette Smoking on the Oral Cavity: A Pilot Study. Dent. J..

[9] Goniewicz M.L., Knysak J., Gawron M., Kosmider L., Sobczak A., Kurek J., Prokopowicz A., Jablonska-Czapla M., Rosik-Dulewska C., Havel C. (2013). Levels of selected carcinogens and toxicants in vapour from electronic cigarettes. Tob. Control.

[10] Cheng T. (2014). Chemical evaluation of electronic cigarettes. Tob. Control.

[11] Irusa K.F., Vence B., Donovan T. (2020). Potential oral health effects of e-cigarettes and vaping: A review and case reports. J. Esthet. Restor. Dent. Off. Publ. Am. Acad. Esthet. Dent..

[12] Cantatore F.P., Corrado A., Covelli M., Lapadula G. (2000). Lo studio morfologico del microcircolo nelle connettiviti [Morphologic study of the microcirculation in connective tissue diseases]. Ann. Ital. Di Med. Interna Organo Uff. Della Soc. Ital. Di Med. Interna.

[13] Lova R.M., Miniati B., Macchi C., Gulisano M., Gheri G., Catini C., Conti A.A., Gensini G.F. (2002). Morphologic changes in the microcirculation induced by chronic smoking habit: A videocapillaroscopic study on the human labial mucosa. Am. Heart J..

[14] Cooke J.P., Bitterman H. (2004). Nicotine and angiogenesis: A new paradigm for tobacco-related diseases. Ann. Med..

[15] Lee J., Taneja V., Vassallo R. (2011). Cigarette smoking and inflammation: Cellular and molecular mechanisms. J. Dent. Res..

[16] Sundar I.K., Javed F., Romanos G.E., Rahman I. (2016). E-cigarettes and flavorings induce inflammatory and pro-senescence responses in oral epithelial cells and periodontal fibroblasts. Oncotarget.

[17] Scardina G.A., Messina M., Melilli D., Cumbo E., Carini F., Tomasello G., Messina P. (2019). Permanence of Modifications in Oral Microcirculation in Ex-Smokers. Med. Sci. Monit. Int. Med. J. Exp. Clin. Res..

